# Sparse Adaptive Iteratively-Weighted Thresholding Algorithm (SAITA) for $L_{p}$-Regularization Using the Multiple Sub-Dictionary Representation

**DOI:** 10.3390/s17122920

**Published:** 2017-12-15

**Authors:** Yunyi Li, Jie Zhang, Shangang Fan, Jie Yang, Jian Xiong, Xiefeng Cheng, Hikmet Sari, Fumiyuki Adachi, Guan Gui

**Affiliations:** 1 College of Electronic and Optical Engineering & College of Microelectronics, Nanjing University of Posts and Telecommunications, Nanjing 210023, China; 2016020221@njupt.edu.cn (Y.L.); chengxf@njupt.edu.cn (X.C.); 2 College of Telecommunication and Information Engineering, Nanjing University of Posts and Telecommunications, Nanjing 210023, China; b13011409@njupt.edu.cn (J.Z.); sponder@126.com (S.F.); jyang@njupt.edu.cn (J.Y.); jxiong@njupt.edu.cn (J.X.); hikmet@njupt.edu.cn (H.S.); 3 Research Organization of Electrical Communication, Tohoku University, Sendai 980-8577, Japan; adachi@ecei.tohoku.ac.jp

**Keywords:** *L_p_*-norm regularization, adaptive weighted, iterative thresholding, multiple dictionaries, single–dictionary, image restoration

## Abstract

Both $L_{1/2}$ and $L_{2/3}$ are two typical non-convex regularizations of $L_{p}$ (0<p<1), which can be employed to obtain a sparser solution than the $L_{1}$ regularization. Recently, the multiple-state sparse transformation strategy has been developed to exploit the sparsity in $L_{1}$ regularization for sparse signal recovery, which combines the iterative reweighted algorithms. To further exploit the sparse structure of signal and image, this paper adopts multiple dictionary sparse transform strategies for the two typical cases p∈{1/2, 2/3} based on an iterative $L_{p}$ thresholding algorithm and then proposes a sparse adaptive iterative-weighted $L_{p}$ thresholding algorithm (SAITA). Moreover, a simple yet effective regularization parameter is proposed to weight each sub-dictionary-based $L_{p}$ regularizer. Simulation results have shown that the proposed SAITA not only performs better than the corresponding $L_{1}$ algorithms but can also obtain a better recovery performance and achieve faster convergence than the conventional single-dictionary sparse transform-based $L_{p}$ case. Moreover, we conduct some applications about sparse image recovery and obtain good results by comparison with relative work.

## 1. Introduction

Compressed sensing (CS) [1,2] and sparse representation [3,4] have been widely used in the field of wireless communications [5,6,7] and image processing [8,9,10]. CS implies that it is possible to reconstruct the sparse signal/image from incomplete data if some prior knowledge and reconstruction constraints are satisfied. Mathematically, the unconstrained $L_{0}$ minimization is the optimal model to obtain the sparsest solution ${\hat{x}}_{l_{0}}$:

(1) $${\hat{x}}_{l_{0}}=\arg\;\underset{\hat{x}\;}{\min}\;\{\gamma {∥y-\Phi x∥}_{2}^{2}+\lambda {∥x∥}_{0}\},$$

where $∥x{∥}_{0}$ denotes the zero-norm function to find the number of nonzero elements in x; $\Phi \in R^{M\times N}$ denotes the down-sampling measurement matrix; y and x represent the observed vector and the unknown sparse image, respectively; λ is the regularization parameter to balance between the fidelity of the image and the sparsity; and γ>0 is a small positive constant, e.g., γ=1/2. Unfortunately, this problem (1) is an NP (non-deterministic) problem, and thus, it is difficult to efficiently solve. When the matrix Φ satisfies some necessary conditions [11], an alternative convex relaxation method are developed using the $L_{1}$ regularization method as:

(2) $${\hat{x}}_{l_{p}}=\arg\;\underset{\hat{x}\;}{\min}\;\{\gamma {∥y-\Phi x∥}_{2}^{2}+\lambda {∥x∥}_{1}\},$$

where $∥x{∥}_{1}=\sum_{i=1}^{n}\left|x_{i}\right|$ denotes the $L_{1}$-norm. Then, the NP problem (1) is converted into problem (2), which is a typical a convex optimization problem and can be solved efficiently, such as with the alternating direction method of multipliers (ADMM) [12,13], fast iterative shrinkage-thresholding algorithm (FISTA) [14], Nesterov’s algorithm (NESTA) [15], and approximate message passing (AMP) [16]. However, the method of $L_{1}$ regularization can only obtain a suboptimal solution and usually requires much more measurements. Theoretical analysis of CS implies that better performance can be obtained by taking advantage of sparser information in many systems, especially in the presence of strong noise interference.

### 1.1. The Non-Convex Penalties

Many state-of-the-art algorithms have been proposed to improve the performance of the $L_{1}$ regularization algorithms. The non-convex penalty regularization algorithms are among the most effective algorithms for sparse recovery problems. Research shows that the non-convex penalty-based optimization methods can more closely approximate the sparsest solution over the $L_{1}$-norm penalty in problem (2), which requires a weaker incoherent condition and fewer measurement data [17]. There have been many non-convex functions proposed as relaxations of the $L_{0}$-norm penalty, such as the smoothly clipped absolute deviation penalty (SCAD) [18], the $L_{p},\;(0<p<1)$-norm penalty [17] and the minimax concave penalty (MCP) [19]. By replacing the $L_{1}$-norm with the $L_{p}$-norm, the non-convex $L_{p}$-norm regularization optimization method is described as:

(3) $${\hat{x}}_{l_{p}}=\arg\;\underset{\hat{x}\;}{\min}\;\{\gamma {∥y-\Phi x∥}_{2}^{2}+\lambda {∥x∥}_{p}^{p}\},\;0<p<1,$$

where $∥x{∥}_{p}^{p}=\overset{n}{\underset{i=1}{\sum }}{\left|x_{i}\right|}^{p}$. Unfortunately, when 0<p<1, problem (3) becomes a non-convex, non-smooth, and non-Lipschitz optimization problem. Thus, the $L_{p}$-norm optimization is always difficult to efficiently address. 

### 1.2. The Iterative Thresholding Algorithm of $L_{p}$ Regularization

There are two main classes of algorithms to solve the non-convex $L_{p}$-norm optimization problem. One is the iteratively reweighted algorithm [20], and the other is the iterative thresholding algorithm (ITA). As one of the most effective and efficient methods, the ITA has been employed for many sparse recovery optimization problems due to its low computational complexities, including the iterative hard thresholding for $L_{0}$ regularization [21], the iterative soft thresholding for $L_{1}$ regularization [22] and the iterative $L_{p}$ thresholding for $L_{p}$ regularization [23]. $L_{1/2}$ and $L_{2/3}$ regularizations are two special and important cases, not only their solutions can be expressed in closed-forms, but also their importance on sparse modeling. Recent studies show that $L_{1/2}$ regularizer can be taken as the most representative $L_{p}$ regularizer [24] and $L_{2/3}$ regularization is more effective in image deconvolution problem [25]. Hence, in this paper, we focus on the $L_{1/2}$ and $L_{2/3}$ regularizations, which is described in Equation (4) and (5):

(4) $${\hat{x}}_{l_{1/2}}=\arg\;\underset{\hat{x}\;}{\min}\;\{\gamma {∥y-\Phi x∥}_{2}^{2}+\lambda {∥x∥}_{1/2}^{1/2}\},$$

(5) $${\hat{x}}_{l_{2/3}}=\arg\;\underset{\hat{x}\;}{\min}\;\{\gamma {∥y-\Phi x∥}_{2}^{2}+\lambda {∥x∥}_{2/3}^{2/3}\},$$

### 1.3. New Multiple-State Sparse Transform Based $L_{1}$ Regularization Algorithm

Recently, some new multiple-state sparse transform based algorithms were proposed to exploit more a priori knowledge of the signal/image by employing some new sparsifying transform strategies. A shearlet-based multiple level sparse representation algorithmic framework was proposed in [26,27] for the unconstrained $L_{1}$ regularization by adaptively incorporating the iteratively reweighted shrinkage step. To enhance the sparsity, the algorithm [27] is specifically adapted to the sparse structure of the multiscale coefficients based on the ADMM [12,13]. Similarly, considering the fact that the sparsity of a certain signal/image will change under different sparsifying transform dictionaries, a sparsity-induced composite regularization algorithm was proposed for the unconstrained $L_{1}$ regularization problem (Co-L1) [28]. The novel Co-L1 method is described as:

(6) $${\hat{x}}_{l_{d,1}}^{t}=\arg\;\underset{\hat{x}\;}{\min}\{{∥\Phi x-y∥}_{2}^{2}+\sum_{d=1}^{D}\lambda_{d,1}{∥\Psi_{d}x∥}_{1}\},$$

where $\Psi_{d}=[\psi_{1}|\psi_{2}\left|\cdots \right|\psi_{N_{d}}]\in R^{N_{d}\times N},\;\left(d=1,\cdots ,D\right)$ are different dictionaries, d denotes the number of $\Psi_{d}$, and $N_{d}$ represents the dictionary size. The regularization parameters $\lambda_{d,1}=\frac{N_{d}}{\varepsilon +{∥\Psi_{d}x∥}_{1}}$ play the roles of weighting the $L_{1}$-norm of the sparsifying transform coefficients $\Psi_{d}x$. Multiplying by the weighting parameter $\lambda_{d,1}^{t}={\left[\frac{N_{1}}{\varepsilon +{∥\Psi_{1}x∥}_{1}},\;\cdots ,\frac{N_{d}}{\varepsilon +{∥\Psi_{d}x∥}_{1}}\right]}^{T}$, the regularizer $\sum_{d=1}^{D}\lambda_{d,1}{∥\Psi_{d}x∥}_{1}^{1}$ is indeed a composition of multiple dictionary based regularizers. The algorithm [28] can significantly improve the image reconstruction performance over the fast iterative shrinkage-thresholding algorithm (FISTA) [29] by iteratively and adaptively weighting the composite regularizer. We define these new emerged sparsifying transforms as the “multiple-state” transform. The common property of these methods is how to exploit the prior information from the multiple-state sparsifying transform to improve the conditioning of the sparse recovery problems.

In this paper, benefiting from the improvement of the $L_{p}$ regularization algorithm [24,25,30], and motivated by recent advances in the iterative reweighted algorithms, we propose a new iteratively-weighted algorithm framework for $L_{p},\;p\in \left\{1/2,\;2/3\right\}$, norm minimization using the multiple-state sparsifying transform, i.e., multiple sub-dictionary sparse representation [28]. The contributions of this paper are summarized as follows. (1) Based on the multiple sub-dictionary sparse representation, we develop a new iteratively-weighted $L_{p}\left(p\in \left\{1/2,\;2/3\right\}\right)$ thresholding algorithm, which is called as SAITA-$L_{p}\left(p\in \left\{1/2,\;2/3\right\}\right)$. (2) By comparison with the existing iteratively-reweighted parameter scheme, we propose an updating regularization parameter for weighting the sub-dictionary. (3) $L_{1/2}$ regularization and $L_{2/3}$ regularization are special and important on sparse modeling, particularly on sparse recovery. However, related studies are rare, this paper also extend the applications to sparse image recovery and Magnetic resonance imaging (MRI) and get good results. 

The organization of the rest of the paper is as follows: in Section 2, we propose the multiple sub-dictionary $L_{p}$-regularization in the SAITA-$L_{p}$ algorithm, including the multiple sub-dictionary sparsifying transforms and the iterative reweighted scheme for the SAITA-$L_{p}$ regularizer. Then, in Section 3, we develop a new $L_{p}\;$ norm minimization, iteratively thresholding algorithm SAITA-$L_{p}$. To confirm the proposed algorithm, we conduct simulations and applications in image restorations in Section 4. In Section 5, we further validate our proposed algorithm using three applications. Finally, conclusions are given in Section 6.

## 2. The Proposed Multiple Sub-Dictionary-Based $L_{p}$ Regularization

The multiple dictionary sparsifying transform method for the $L_{1}$ regularization optimization problem was proposed in [28], which extends the well-known Lasso problem into a composite regularization problem. Motivated by the composite regularization method for the $L_{1}$-norm, this paper employs the multiple sub-dictionary method for the $L_{p}$ regularization problem.

Suppose $x\in R^{N\times 1}$ is the non-sparse, raw signal. We can obtain the sparse coefficients Ψx through an analysis dictionary $\Psi \in R^{N_{1}\times N}$. The shearlet transform [31] and the wavelet transform [32] are two typical sparsifying transforms. We choose the wavelet transform as the ideal transform because of its effectiveness to compress natural images. The main steps to design the multiple sub-dictionary sparsifying transform based $L_{p}$ regularization method are:

First, we construct an (DN)×N over-complete sparsifying transform matrix Ψ by:

(7) $$\Psi =\left[\begin{array}{c} \Psi_{1}\\ \vdots \\ \Psi_{d}\\ \vdots \\ \Psi_{D} \end{array}\right]\in R^{\left(DN\right)\times N},$$

and:

(8) $$\Psi_{d}=\left[\begin{array}{c} \psi_{1}\\ \psi_{2}\\ \vdots \\ \psi_{N_{d}} \end{array}\right]\in R^{N_{d}\times N},$$

where D denotes the number of sub-dictionaries $\Psi_{d}$ in the over-complete sparsifying transform matrix Ψ, the $N_{d}\times N$ sub-dictionary matrix $\Psi_{d},\;\left(d=1,\cdots ,D\right)$ is acquired by a collection of row vectors ${\left\{\psi_{i}\right\}}_{i=1}^{N_{d}}$, such as the “*dbN*” wavelet basis [33], and $N_{d}$ represents the number of column in $\psi_{i}$. From Equation (8) we can see that each $\Psi_{d}$ is composed of a set of rows from the (DN)×N over-complete sparsifying transform matrix Ψ, and:

(9) $$N_{1}+N_{2}+\cdots +N_{d}=DN,$$

By splitting the matrix Ψ into several sub-dictionaries $\Psi_{d}$, we convert the sparsifying transform Ψx to a composition of several $\Psi_{d}x,\;d=1,2,\cdots ,D$ with different sparse structures. Intuitively, we can utilize these differences to improve the recovery performance in sparse recovery problems. In this paper, we choose $N_{1}=N_{2}=\cdots =N_{d}=N$, so $\Psi_{d}\in R^{N\times N}$, which are orthogonal matrixes.

Then, a new multiple non-convex $L_{p}$ regularization method is proposed:

(10) $${\hat{x}}_{l_{d,p}}^{t}=\arg\;\underset{\hat{x}\;}{\min}\left\{f_{d}\left(x\right)=\gamma {∥\Phi x-y∥}_{2}^{2}+R_{SAITA}\right\},$$

where $R_{SAITA}$ is a linearly weighted combination of multiple sub-dictionary based $L_{p}$ regularizers ${∥\Psi_{d}x∥}_{p}^{p}$:

(11) $$R_{SAITA}=\sum_{d=1}^{D}\lambda_{d,p}^{t}{∥\Psi_{d}x∥}_{p}^{p}=\lambda_{1,p}^{t}{∥\Psi_{d}x∥}_{p}^{p}+\lambda_{2,p}^{t}{∥\Psi_{d}x∥}_{p}^{p}+\cdots +\lambda_{D,p}^{t}{∥\Psi_{d}x∥}_{p}^{p},$$

the $\lambda_{d,p},\;d=1,2,\cdots D$ denotes the iterative weighted regularization parameter. Hence, the contribution of each sub-dictionary is controlled adaptively and iteratively with the weighted parameter $\lambda_{d,p}$, and the regularizer ${∥\Psi_{d}x∥}_{p}^{p}$ will vary across the sub-dictionary index d. Intuitively, the variation of each sub-dictionary based regularizer is best weighted by the parameter $\lambda_{d,p}$ to improve the sparse recovery problem.

## 3. The Proposed SAITA-$L_{p}$ Algorithm

The major disadvantage of the $L_{p}(0<p<1)$ minimization is that it is nonconvex, making it difficult to efficiently solve. In this section, we first introduce the iteratively thresholding representation theory for the conventional $L_{p},\left(p\in \left\{1/2,\;2/3\right\}\right)$ algorithm according to the existing series of algorithms in [25,34]. Then, we deduce the SAITA-$L_{p}$ algorithm combined with the proposed weighted scheme $\lambda_{d,p}$.

### 3.1. The Relationship of the SAITA-$L_{p}$ and Conventional $L_{p}$ Methods

Considering the multiple sub-dictionary $L_{p},\left(p\in \left\{1/2,\;2/3\right\}\right)$ regularization problem in Equation (10), when γ=1, we have:

(12) $${\hat{x}}_{l_{d,p}}=\arg\;\underset{\hat{x}\;}{\min}\left\{f_{d}\left(x\right)={∥\Phi x-y∥}_{2}^{2}+\sum_{d=1}^{D}\lambda_{d,p}{∥\Psi_{d}x∥}_{p}^{p}\right\},$$

where the $f_{d}\left(x\right)$ denotes the objective functions. Correspondingly, the conventional single dictionary $\Psi^{\prime }\in R^{N\times N}$ based analysis $L_{p}$-norm minimization problem is as follows:

(13) $${\hat{x}}_{l_{p}}^{t}=\arg\;\underset{\hat{x}\;}{\min}\left\{f\left(x\right)={∥\Phi x-y∥}_{2}^{2}+\lambda_{p}{∥\Psi^{\prime }x∥}_{p}^{p}\right\},$$

**Figure 1:**
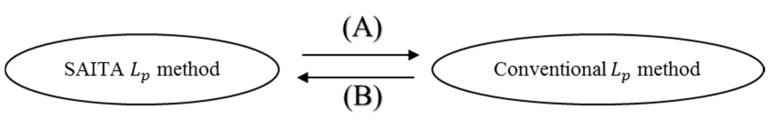
The relationship between the conventional single dictionary based $L_{p}$ thresholding method and the proposed SAITA $L_{p}$ method.

The proposed SAITA-$L_{p}\left(p\in \left\{1/2,\;2/3\right\}\right)$ methods of (12) and the conventional method (13) are nearly identical, and the major difference is how to weight the contribution of the $L_{p}$-norm by the regularization parameter [28]. Compared with the conventional method, the SAITA method can exploit more prior knowledge of the sparse signal/image for reconstruction. Figure 1 depicts the relationship between the two methods. In the case of (A), the number of sub-dictionaries d is reduced to 1, and the multiple dictionaries $\Psi_{d}$. convert into a single Ψ. Then, the proposed SAITA-$L_{p}$ algorithm converts to the conventional single dictionary $L_{p}$ method [24,25]. In case (B), with the increase of the number d, the conventional single dictionary $L_{p}$ method converts to the proposed SAITA-$L_{p}$ method.

### 3.2. The Thresholding Representation Theory for SAITA-L_p_

According to the relationship between the proposed SAITA-$L_{p}$ method and the conventional $L_{p}$ method shown in Figure 1, we first consider the conventional single dictionary analysis $L_{p}$ problem (13). The first order optimality condition of x is described as:

(14) $$\nabla f\left(x\right)=2\Psi^{\prime }\Phi^{T}\left(\Phi x-y\right)+\lambda \nabla ({∥\Psi^{\prime }x∥}_{p}^{p})$$

in which the operator ∇(·) denotes the gradient. Letting ∇f(x)=0, we have:

(15) $$\Psi^{\prime }\Phi^{T}\left(y-\Phi x\right)=\frac{1}{2}\left(\lambda \nabla \left({∥\Psi^{\prime }x∥}_{p}^{p}\right)\right),$$

Multiplying by any parameter τ to control the step size and adding $\Psi^{\prime }x$ in both sides of Equation (15):

(16) $$\Psi^{\prime }x+\tau \Psi^{\prime }\Phi^{T}\left(y-\Phi x\right)=\Psi^{\prime }x+\frac{1}{2}\left(\lambda \tau \nabla \left({∥\Psi^{\prime }x∥}_{p}^{p}\right)\right),$$

Then, we can immediately obtain:

(17) $$\left(I+\frac{\lambda \tau }{2}\nabla ({∥\;\cdot \;∥}_{p}^{p}\right)\Psi^{\prime }x=\Psi^{\prime }x+\tau \Psi^{\prime }\Phi^{T}\left(y-\Phi x\right)$$

That is:

(18) $$\Psi^{\prime }x={\left(I+\frac{\lambda \tau }{2}\nabla ({∥\;\cdot \;∥}_{p}^{p}\right)}^{-1}\Psi^{\prime }\left(x+\tau \Phi^{T}\left(y-\Phi x\right)\right)$$

To this end, when p∈{1/2, 2/3}, the resolvent operator [24,25,30] is denoted as:

(19) $$H_{\lambda ,p}\left(\cdot \right)={\left(I+\frac{\lambda \tau }{2}\nabla ({∥\;\cdot \;∥}_{p}^{p}\right)}^{-1},$$

where λ and τ are the regularization parameter and the step tunning parameter, respectively. Then:

(20) $$\Psi^{\prime }x=H_{\lambda ,p}\left(\Psi^{\prime }\left(x+\tau \Phi x^{T}\left(y-\Phi x\right)\right)\right)$$

where τ>0 (e.g., $\tau =\frac{0.99}{{∥\Phi ∥}_{2}^{2}}$, or $\tau =\frac{0.99}{{∥\Phi ∥}_{2}}$) controls the step size in each iteration. 

According to the Equation (20), we immediately imply:

(21) $$x^{n+1}={\left(\Psi^{\prime }\right)}^{-1}H_{\lambda ,p}\left(\theta \left(x^{n}\right)\right),$$

in which:

(22) $$\theta \left(x^{n}\right)=\Psi^{\prime }\left(x^{n}+\tau \Phi^{T}\left(y-\Phi x^{n}\right)\right),$$

where $x^{n}$ represents the n-th iterative solution. The resolvent operator $H_{\lambda ,p}\left(\cdot \right)$ is defined as:

(23) $$H_{\lambda ,p}\left(x\right)={\left(h_{\lambda ,p}\left(x_{1}\right),h_{\lambda ,p}\left(x_{2}\right),\cdots ,h_{\lambda ,p}\left(x_{N}\right)\right)}^{T},$$

where the $h_{\lambda ,p}\left(x_{i}\right)$ is the nonlinear function defined by:

(24) $$h_{\lambda ,p}\left(x_{i}\right)=\{\;\begin{array}{c} \varphi_{\lambda_{d,p}}\left(x_{i}\right),\;\left|x_{i}\right|>T\\ \;0,\;otherwise \end{array},$$

when $p=\frac{1}{2}$; $T=\frac{3\sqrt[{3}]{2}}{4}{\left(\lambda_{d,1/2}\tau \right)}^{2/3}$ is the threshold value, and [24]:

(25) $$\varphi_{\lambda ,1/2}\left(x_{i}\right)=\frac{2}{3}x_{i}\left(1+\cos\left(\frac{2\pi }{3}-\frac{2}{3}cos^{-1}\left(\frac{\lambda_{d,1/2}\tau }{8}{\left(\frac{\left|x_{i}\right|}{3}\right)}^{-\frac{3}{2}}\right)\right)\right),$$

when $p=\frac{2}{3}$; $T=\frac{2\sqrt[{4}]{3}}{3}{\left(\lambda_{d,2/3}\tau \right)}^{3/4}$ is the related threshold value, and [25]:

(26) $$\varphi_{\lambda ,2/3}\left(x_{i}\right)={\left(\frac{\vartheta_{\lambda ,2/3}\left(x_{i}\right)+\sqrt{\frac{2\left|x\right|}{\left|\vartheta_{\lambda ,2/3}\left(x_{i}\right)\right|}-{\left|\vartheta_{\lambda_{d,2/3}}\left(x_{i}\right)\right|}^{2}}}{2}\right)}^{3}\cdot \mathrm{sgn}\left(t\right),$$

where sgn(⋅) denotes the sign function and

(27) $$\vartheta_{\lambda_{d,2/3}}\left(x_{i}\right)=\frac{2}{\sqrt{3}}{\left(\lambda_{d,2/3}\tau \right)}^{1/4}{\left(\cos h\left(\frac{1}{3}arccosh\left(\frac{27}{16}{\left(\lambda_{d,2/3}\tau \right)}^{-3/2}x_{i}{}^{2}\right)\right)\right)}^{1/2}$$

### 3.3. The Proposed SAITA-L_p_ Algorithm

As mentioned above, the iteratively weighted parameter plays a key role during the optimization process for the sparse recovery problem. For the proposed SAITA-*L_p_* method, the iteratively weighted parameter $\lambda_{d,p}$ mainly plays two roles. One is the role of controlling the tradeoff of the fidelity and the prior knowledge between the quadratic term and the regularizer, and the other role is controlling the contribution of each regularizer. Unfortunately, it is not clear how to do this because setting an ideal parameter is not straightforward. In [28], a iteratively updating parameter was reduced by applying a Maximization-Minimization algorithm shown as:

(28) $$\lambda_{d,1}=\frac{N_{d}}{\epsilon +{∥\Psi_{d}x∥}_{1}^{1}}$$

where $N_{d}$ controls the sub-dictionary size, ϵ>0 is a small constant which prevent the denominator form zero. From Equation (28) we can obtain some useful information about setting a proper regularization parameter. Firstly, the contribution of denominator in Equation (28) is to counterweigh each sub-dictionary based regularizer; Secondly, the numerator $N_{d}$ control the size of each sub-dictionary. Inspired by the above insights, in this paper, we design the important iteratively weighted parameter $\lambda_{d,p}$ as:

(29) $$\lambda_{d,p}=\frac{N_{d}}{{\left(\epsilon +{∥\Psi_{d}x∥}_{2}^{2}\right)}^{\alpha }},$$

where $N_{d}$ controls the sub-dictionary size as showed in [28], α∈(0,2) is a small constant to tune it that is determined from the following experimental results. Then the parameter $\lambda_{d,p}^{t}$ plays the role of weighting each $L_{p}$-norm regularizer adaptively.

The following are the analytical justifications. (i) When signal x is sparser under any dictionary of $\Psi_{d}$ than others, the value of each regularizer ${∥\Psi_{d}x∥}_{p}^{p}$ is smaller. Hence, the dictionary of $\Psi_{d}$ is more appropriate, and on the other hand, a smaller regularizer ${∥\Psi_{d}x∥}_{p}^{p}$ will be beneficial to minimizing the objective function. Thus, the weight of the regularizer should be enhanced. (ii) When the signal x may not be sparse enough under another dictionary $\Psi_{d}$, that is, the dictionary of $\Psi_{d}$ is not ideal, the value of ${∥\Psi_{d}x∥}_{p}^{p}$ will be larger. The larger ${∥\Psi_{d}x∥}_{p}^{p}$ will not be helpful to minimizing the objective function; thus the weight of the ${∥\Psi_{d}x∥}_{p}^{p}$ should be smaller to counterweigh the regularizer.

For the main comparison, the conventional single-dictionary based $L_{p},\;p\in \left\{1/2,\;2/3\right\}$-regularization method in problem (13) will be considered, and the tradeoff parameter $\lambda_{p}$ is a fixed constant, which is shown as:

(30) $$\lambda_{p}=\frac{1}{{\left(\epsilon +{∥\Psi x∥}_{2}^{2}\right)}^{\alpha }}.$$

From Equation (30), we find that the conventional single-dictionary based parameter $\lambda_{p}$ is a constant and will not vary. 

Moreover, we employ the forward-backward linear strategy to accelerate the convergence of the proposed algorithm as [14]:

(31) $$\mu^{n+1}=\frac{1+\sqrt{1+4{\left(\mu^{n}\right)}^{2}}}{2},$$

and:

(32) $$x^{n+1}=x^{n}+\frac{\mu^{n}-1}{\mu^{n+1}}\left(x^{n}-x^{n-1}\right),$$

The proposed iteratively-weighted SAITA-$L_{p}$ algorithm can be described in Algorithm 1.
**Algorithm 1**: The proposed SAITA-$L_{p}$ algorithm.  **Problem: **$x^{n+1}=\arg\min\gamma {∥\Phi x-y∥}_{2}^{2}+\sum_{d=1}^{D}\lambda_{d,p}{∥\Psi_{d}x∥}_{p}^{p}$;  **1: Input:**${\left\{\Psi_{d}\right\}}_{d=1}^{D}$, y, Φ; $L_{d}$; γ=1; ϵ>0;  **2: Initialization:**
n=0; ε=0.01; $\tau =\frac{1-\varepsilon }{{∥\Phi ∥}^{2}}$; $\lambda_{d,1/2}^{\left(1\right)}=1$; α.  **3: for**
n=1, 2, 3, ⋯  **4: Calling the conventional analysis**
$L_{p}$
**algorithm in (13):**   **While not converged do**    Step 1: Compute $\theta \left(x^{n}\right)=\Psi^{\prime }\left(x^{n}+\Phi^{T}\left(y-\Phi x^{n}\right)\right)$ in Equation (22);    Step 2: Compute $x^{n+1}={\left(\Psi^{\prime }\right)}^{-1}H_{\lambda ,p}\left(\theta \left(x^{n}\right)\right)$ in Equation (21);    Step 3: Update the value of μ using $\mu^{n+1}=\frac{1+\sqrt{1+4{\left(\mu^{n}\right)}^{2}}}{2}$ in Equation (31);    Step 4: Update the solution $x^{n+1}=x^{n}+\frac{\mu^{n}-1}{\mu^{n+1}}\left(x^{n}-x^{n-1}\right)$ in Equation (32);   **End**  **5: Updating:**
$\lambda_{d,p}^{\left(n+1\right)}\leftarrow \frac{N_{d}}{{\left(\epsilon +{∥\Psi_{d}x^{\left(n\right)}∥}_{2}^{2}\right)}^{\alpha }}$ in Equation (29);  **6: end**  **7: Output**
$x^{t}$

## 4. Performance Analysis and Discussion

We first conduct some experiments to determine the value of α and verify the performance of the proposed $\lambda_{d,p}^{t}$, then we evaluate the superiority of the proposed SAITA algorithm compared with the conventional single dictionary analysis $L_{p}$ iterative thresholding algorithms [24,25] and the Co-L1 [28]. All the experiments in this paper were conducted on a personal computer (2.21 GHz, 16 GB RAM) in a MATLAB (R2014a) platform.

Assuming the clean image x, we construct a measurement matrix Φ using the “Spread Spectrum” operator [35], so the measurement image shows: y=Φx+n, where n denotes the additive noise. Since the wavelet is known to compress natural raw images very efficiently, we choose the wavelet transform as the sparsifying transform operator. Then, we construct the sparsifying transform matrix $\Psi \in R^{8N\times N}$ by concatenating the undecimated ‘db1’ and ‘db2’ wavelet transform with 2-levels of decomposition. Thus, we can obtain the sub-dictionaries: $\Psi_{d}\in R^{N\times N},\;d=1,2,\cdots 8$.

**Figure 2:**
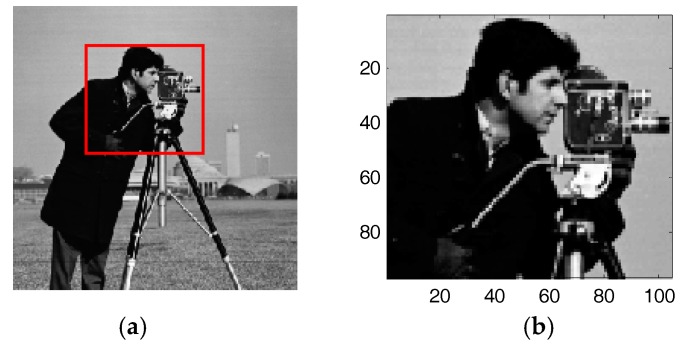
(**a**) the cropped Cameraman image of size N=256×256. (**b**) the selected image portion of size N=96×104.

The SNR measurement is adopted to measure the noise level, which is defined as $mSNR=\frac{{∥y∥}_{2}^{2}}{M\sigma^{2}}$, where M and $\sigma^{2}$ denote the number of y and the variance of the white Gaussian noise, respectively. The higher the value of mSNR, the weaker of the noise level is. We evaluate the performance by the popular recovery SNR: $RSNR=-20\log\left(\frac{{∥x-\hat{x}∥}_{2}}{{∥x∥}_{2}}\right)$, where $\hat{x}$ denotes the estimated sparse image. The higher the value of RSNR, the better the performance. We conduct the experiments by utilizing the well-known figure of “Cameraman” with mSNR=M/N=40 dB, which is shown in Figure 2a. To reduce the computation time, we choose only a part of the figure, shown in Figure 2b.

### 4.1. The Value Range of α in $\lambda_{d,p}$

**Figure 3:**
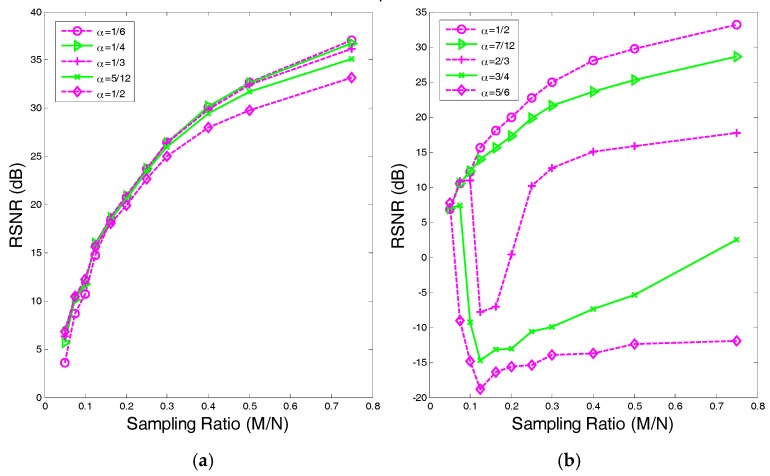
The RSNR of the proposed SAITA-$L_{p}$ algorithm of α∈(0, 2p) and p=1/2. (**a**) The RSNR of $\alpha \in \left\{\frac{1}{6},\frac{1}{4},\frac{1}{3},\frac{5}{12},\frac{1}{2}\right\}$. (**b**) The RSNR of $\alpha \in \left\{\frac{1}{2},\frac{7}{12},\frac{2}{3},\frac{3}{4},\frac{5}{6}\right\}$.

**Figure 4:**
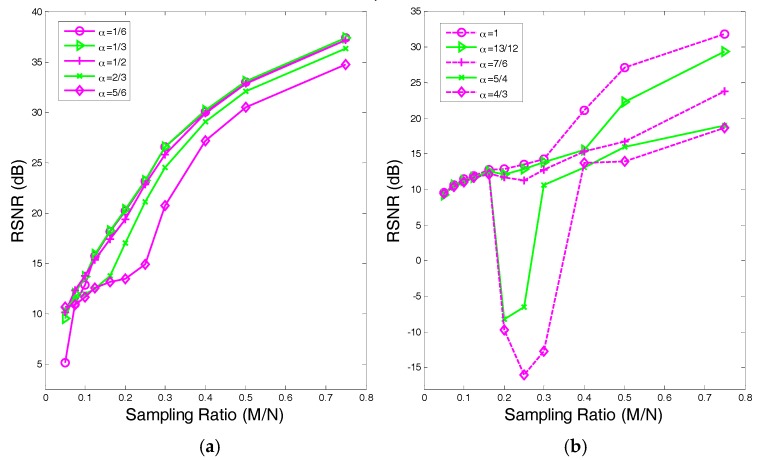
The recovery SNR of the proposed SAITA-$L_{p}$ algorithm of α∈(0, 2p) and p=2/3. (**a**) The RSNR versus Sampling Ratio of $\alpha \in \left\{\frac{1}{6},\frac{1}{3},\frac{1}{2},\frac{2}{3},\frac{5}{6}\right\}$; (**b**) The RSNR versus Sampling Ratio of $\alpha \in \left\{1,\frac{13}{12},\frac{7}{6},\frac{5}{4},\frac{4}{3}\right\}$.

In Section 4.1, we first determine the value range for α in $\lambda_{d,p}$ by evaluating the performances with different values of α. The results are shown in Figure 3 and Figure 4. From the results, we can find that when α∈(0,p), the proposed algorithms perform well and enjoy strong robustness. With the increase of α, the performances deteriorate rapidly. Therefore, we estimate that the value of α should be [0, p] to obtain the adaptive weighting. We specially set:

(33) $$\lambda_{d,p}^{t}=\frac{N_{d}}{{\left(\epsilon +{∥\Psi_{d}x∥}_{2}^{2}\right)}^{\frac{1-p}{2}}},$$

### 4.2. The Recovery SNR Performances Versus Sampling Ratio

**Figure 5:**
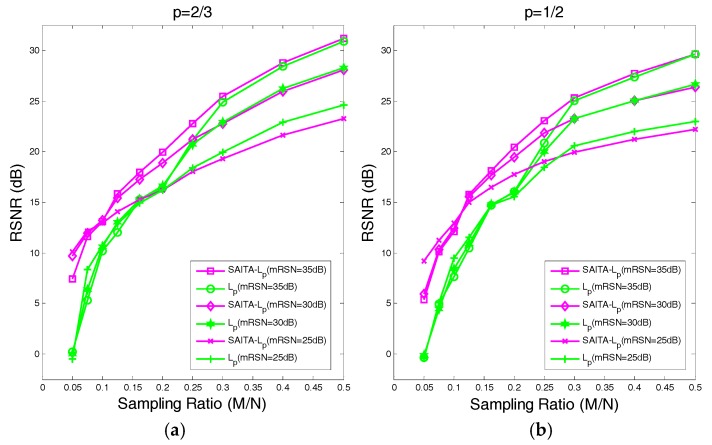
The RSNR performances of the proposed SAITA-$L_{p}$ algorithm and the $L_{p}$ algorithm with mRSN∈{25, 30, 35} dB. (**a**) p=1/2. (**b**) p=2/3.

In Section 4.2, we evaluate the robustness of the proposed algorithm by considering the RSNR versus the sampling ratio. Specifically, we set three mSNR levels to 25 dB, 30 dB and 35 dB. Figure 5 depicts the RSNR versus the sampling ratio. Based on the results, the proposed SAITA-$L_{p},\;\left(p\in \left\{1/2,\;2/3\right\}\right)$ algorithm performs better than the conventional single dictionary based algorithm, and the robustness of the proposed algorithm is good.

### 4.3. The Recovery SNR Performance Versus Measurement SNR

**Figure 6:**
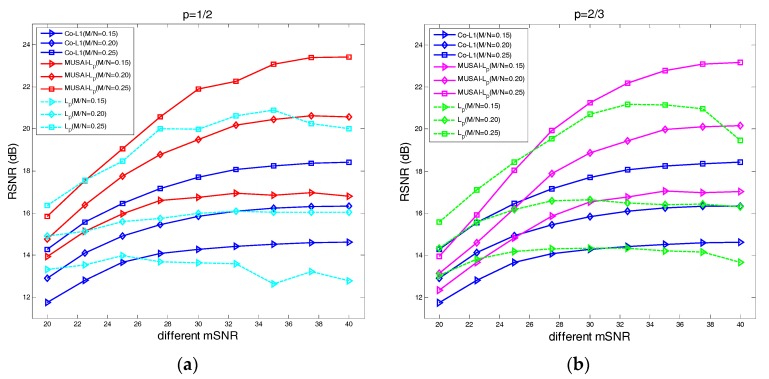
(**a**) The case of p=1/2 for the sampling ratios M/N∈{0.15, 0.20, 0.25} of the Cameraman images. (**b**) The case of p=2/3 for the sampling ratios M/N∈{0.15, 0.20, 0.25} of the cameraman images. The RSNR versus mSNR of the proposed SAITA-$L_{p}\left(p\in \left\{1/2,\;2/3\right\}\right)$ algorithm and the $L_{p}\left(p\in \left\{1/2,\;2/3\right\}\right)$ algorithm for three low sampling ratios M/N∈{0.15, 0.20, 0.25}.

For our third experiment, we investigate the influence of different noise levels on the proposed algorithm and compare the $L_{p}$ algorithm and Co-L1 [28]. Similarly, we consider three sampling ratio levels of M/N∈{0.15, 0.20, 0.25}. We evaluate the performance by the RSNR versus the lower mSNR (20 dB~40 dB) of the image, and the results are presented in Figure 6. 

From the results, we can find that the proposed SAITA-$L_{p},\;\left(p\in \left\{1/2,\;2/3\right\}\right)$ algorithm can obtain a higher recovery SNR and has better robustness and fidelity than the Co-L1. The robustness and fidelity of the corresponding $L_{p},\;\left(p\in \left\{1/2,\;2/3\right\}\right)$ algorithm will deteriorate with the increase of the signal measurement SNR.

### 4.4. The Relative Error Performances Versus the Number of Iterations

We study the convergence and the reconstruction error by the relative error performances versus the number of iterations. Choosing the relative error as the second quality measurement, the formula is given:

(34) $$Relative\;Error=\frac{{∥x-\hat{x}∥}_{2}}{{∥x∥}_{2}}$$

**Figure 7:**
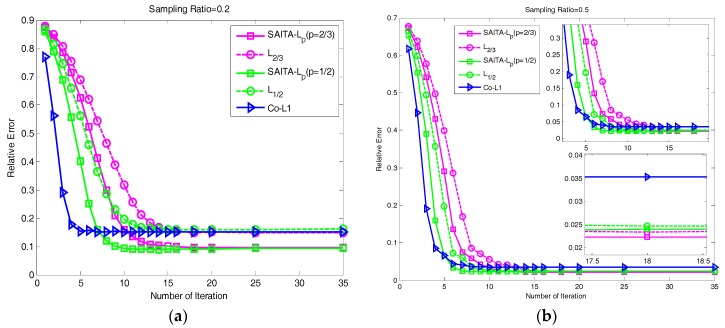
(**a**) The relative error for the lower sampling ratio M/N=0.2 in the cameraman image. (**b**) The relative error for the higher sampling ratio of M/N=0.5 in the cameraman image. The relative errors verse the iteration number of the proposed SAITA-$L_{p}$ algorithm and the $L_{p}$ algorithm.

Considering the proposed SAITA-$L_{p},\;\left(p\in \left\{1/2,\;2/3\right\}\right)$ and the corresponding $L_{p},\;\left(p\in \left\{1/2,\;2/3\right\}\right)$ algorithm from the result shown in Figure 7, when the sampling ratio is 0.2 (shown in (a)), the relative errors of the proposed SAITA-$L_{p},\;\left(p\in \left\{1/2,\;2/3\right\}\right)$ algorithm are significant smaller, and converge faster than the corresponding $L_{p},\;\left(p\in \left\{1/2,\;2/3\right\}\right)$ algorithm. When the sampling ratio is 0.5 (shown in (b)), though the final relative errors are close, the convergence speed of the proposed algorithm is higher (the number of iterations are approximately 15 and 7, respectively). While compared to Co-L1 [28], our proposed SAITA-$L_{p}$ algorithm can obtain a markedly lower relative error when the sampling ratio is M/N∈{0.2, 0.5}. In addition, one can observe that the relative error is slightly smaller than for p=2/3, while the convergence rate is faster than the p=1/2. 

## 5. Practical Experiments

The proposed algorithms can be applied in many practical applications [36,37,38,39,40,41,42]. In this section, we conduct some typical applications about sparse image recovery and medical imaging to extend the applications of $L_{1/2}$ and $L_{2/3}$ regularizations and illustrate the excellent robustness and adaptation of the proposed SAITA-$L_{p}$, (p∈{1/2, 2/3}) algorithm. The conventional single dictionary analysis $L_{p}$ iterative thresholding algorithms [24,25] and the Co-L1 [28] as the standard algorithms for comparison. 

### 5.1. Application 1: Image Sparse Restoration

**Figure 8:**
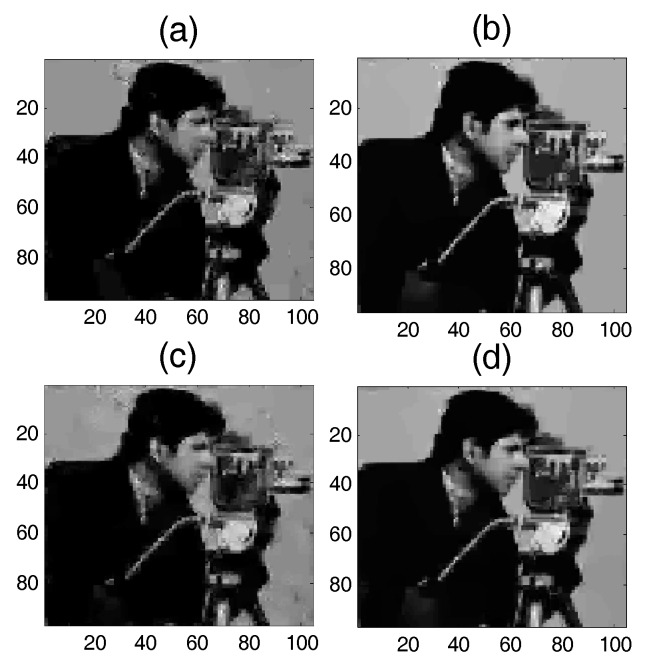
The recovery effects of the proposed SAITA-$L_{p}\left(p\in \left\{1/2,\;2/3\right\}\right)$ and the conventional $L_{p}\left(p\in \left\{1/2,\;2/3\right\}\right)$ algorithms for the M/N=0.2 and mSNR=40 dB cameraman image. (**a**) $L_{1/2}$, RSNR=15.7316 dB; (**b**) SAITA-$L_{p}$ algorithm (p=1/2), RSNR=20.5714 dB; (**c**) $L_{2/3}$, RSNR=16.3098 dB; and (**d**) SAITA-$L_{p}$ algorithm (p=2/3), RSNR=20.1259 dB.

**Figure 9:**
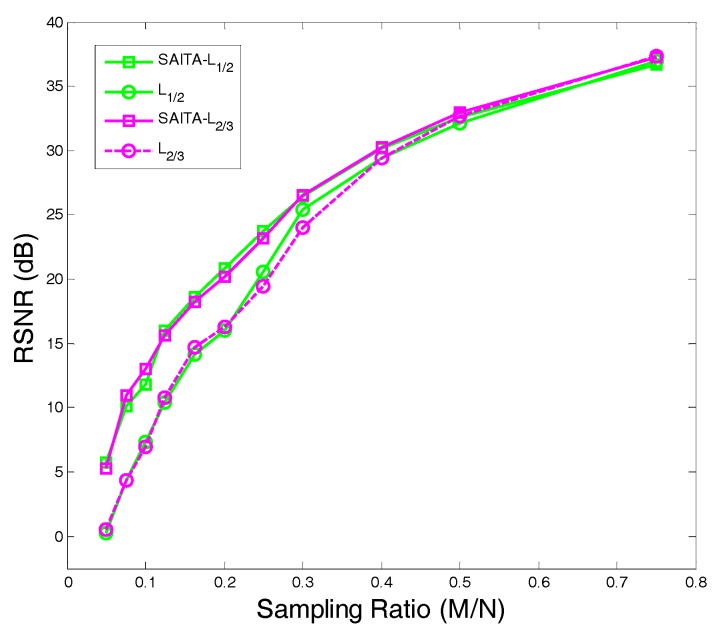
The RSNR of the SAITA-$L_{p}\left(p\in \left\{1/2,\;2/3\right\}\right)$ algorithms vs. the sampling ratio M/N for the mSNR=40 dB cameraman image.

In the first application, the proposed SAITA-$L_{p}$ algorithm is applied to restoring the “Cameraman” image shown in Figure 2 versus sampling ratio M/N. We use the reduced N=96×104 cameraman image as the objective image. Figure 8a,c show the recovery results using the conventional single dictionary algorithm of $L_{1}$ and $L_{p},\left(p\in \left\{1/2,\;2/3\right\}\right)$, respectively. Figure 8b,d show the recovery images using the corresponding multiple sub-dictionary algorithm of $L_{1}$ and $L_{p}\left(p\in \left\{1/2,\;2/3\right\}\right)$, respectively. The experiments show that all the algorithms can recover the images, and the proposed multiple sub-dictionary algorithms significantly outperform the conventional single-dictionary algorithms. In Figure 9, we depict the RSNR of four algorithms vs. different sampling ratios. When p=1/2 and p=2/3, it can be observed that the proposed SAITA algorithm can obtain a lager RSNR compared with the conventional $L_{p}$ algorithms. There is no obvious improvement between the two cases of $p=\frac{1}{2}$ and $p=\frac{2}{3}$, and the SAITA-$L_{1/2}$ algorithm outperforms the SAITA-$L_{2/3}$ algorithm with a weak advantage.

### 5.2. Application 2: Medical Imaging

In the application 2, we extend the applications of our proposed algorithm to solve typical medical construction problems. We first consider the well-known Shepp-Logan phantom, and then we construct a high-quality dMRI cardiac cine [8,28].

#### 5.2.1. Test 1 for the Shepp-Logan Model

In the test 1, we first consider the well-known Shepp-Logan phantom of N=96×96 with an mSNR=40 dB. We construct the compressed noisy measurement signal y by utilizing the “Spread Spectrum” operator as the measurement matrix Φ [35], and we conduct a sparsifying transform with the constructed operator $\Psi \in R^{\left(4N\right)\times N}$ (‘db3’, N=1).

**Figure 10:**
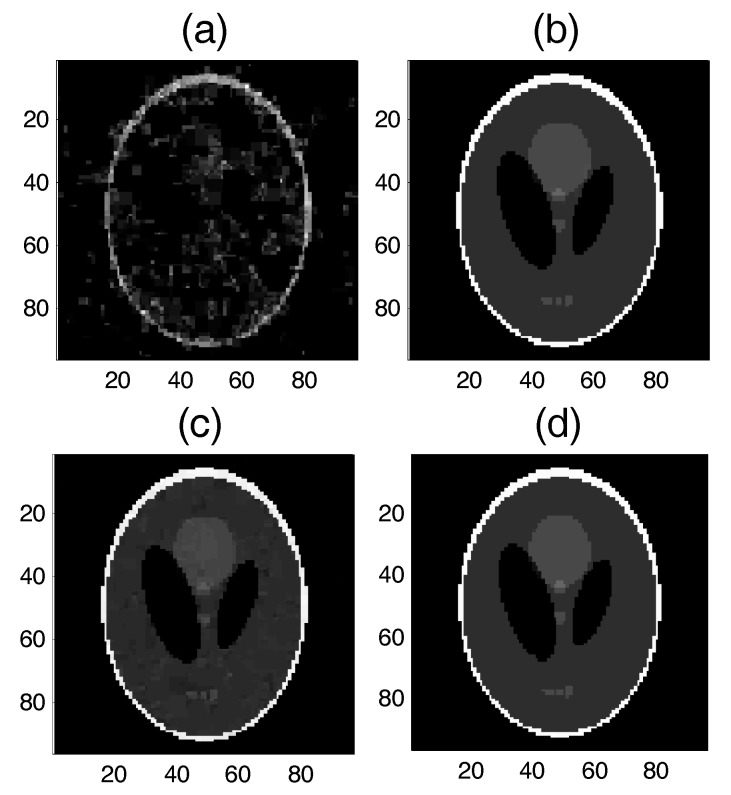
The recovery effects of the proposed SAITA-$L_{p}\left(p\in \left\{1/2,\;2/3\right\}\right)$ and the corresponding $L_{p}\left(p\in \left\{1/2,\;2/3\right\}\right)$ algorithm for the M/N=0.140, mSNR=40 dB Shepp-Logan image. (**a**) $L_{1/2}$, RSNR=3.5436 dB; (**b**) SAITA-$L_{p}$ algorithm (p=1/2), RSNR=43.7016 dB; (**c**) $L_{2/3}$, RSNR=27.2450 dB; and (**d**) SAITA-$L_{p}$ algorithm (p=2/3), RSNR=44.9549 dB.

**Figure 11:**
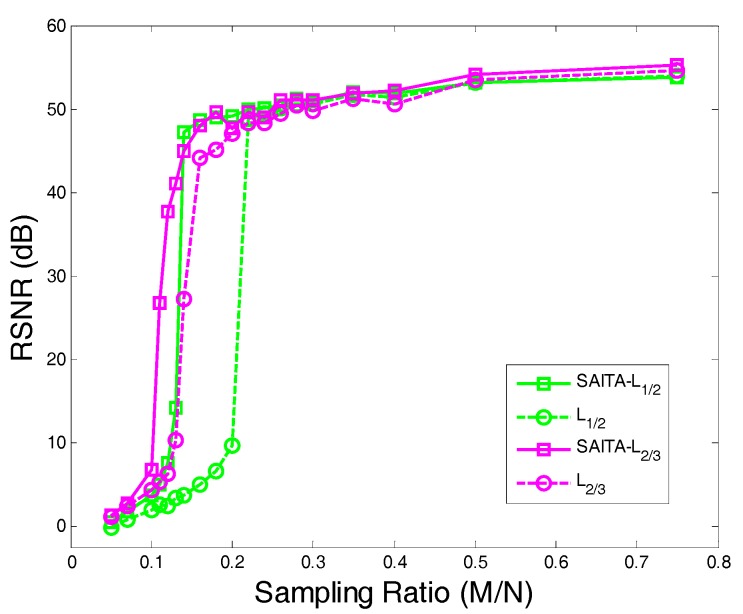
The RSNR of the proposed SAITA-$L_{p}\;\left(p\in \left\{1/2,\;2/3\right\}\right)$ algorithm and the conventional $L_{p}\;\left(p\in \left\{1/2,\;2/3\right\}\right)$ algorithm vs. the sampling ratio M/N for the mSNR=40 dB Shepp-Logan image.

From the experimental results shown in Figure 10, we find that the proposed SAITA-$L_{p}$ algorithm can recover the images perfectly, as shown in Figure 10b,d, while the conventional single dictionary algorithms failed to recover the image, which is shown in Figure 10a,c. The proposed SAITA-$L_{p}$ algorithm of p=2/3 can obtain the best effect compared with the other algorithms, and the next best is the SAITA-$L_{p}$ algorithm of p=1/2. In Figure 11, we depict the RSNR of the respective algorithms versus different sampling ratios M/N. When p=1/2 and p=2/3, it can be observed that the proposed SAITA-$L_{p}$ algorithms can obtain a larger RSNR than the $L_{p}$ algorithms with M/N∈(0.1, 0.2) significantly. 

#### 5.2.2. Test 2 for Real-World Data (2D MRI)

**Figure 12:**
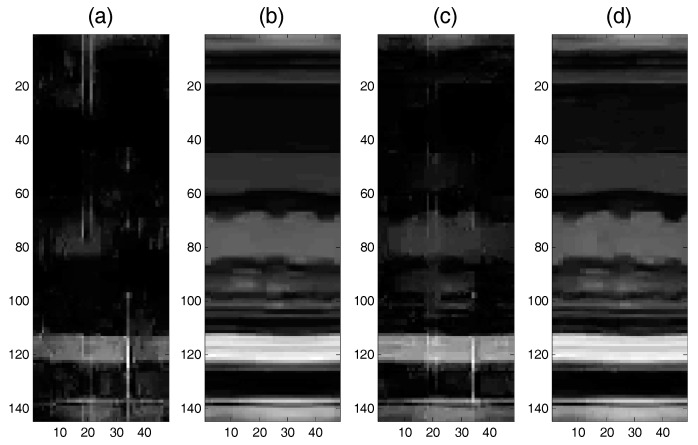
The recovery effects of the proposed SAITA-$L_{p}\left(p\in \left\{1/2,\;2/3\right\}\right)$ and the corresponding $L_{1/2}$ and $L_{2/3}$ algorithm for the M/N=0.2, mSNR=40 dB 2D MRI image. (**a**)$\;L_{1/2}$ algorithm, RSNR=3.6346 dB; (**b**) SAITA-$L_{1/2}$ algorithm, RSNR=21.0189 dB; (**c**) $L_{2/3}$ algorithm, RSNR=7.7718 dB; and (**d**) SAITA-$L_{2/3}$ algorithm, RSNR=23.1872 dB.

**Figure 13:**
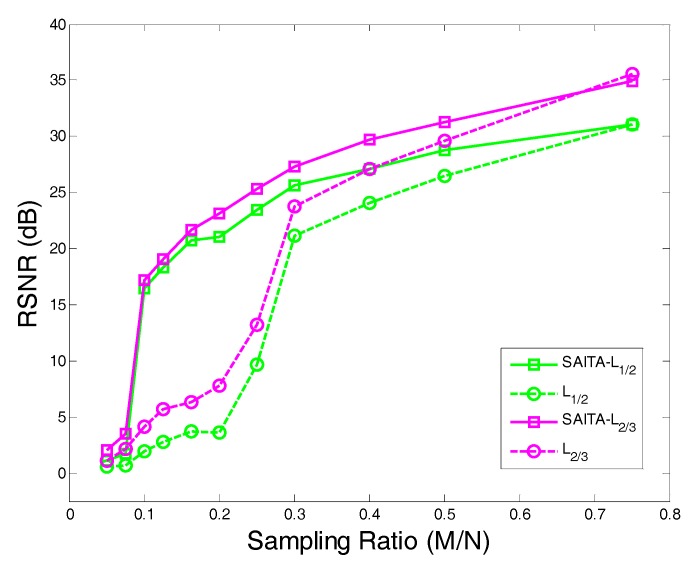
The RSNR of the proposed SAITA-$L_{p},\;\left(p\in \left\{1/2,\;2/3\right\}\right)$ algorithm and the $L_{p},\left(p\in \left\{1/2,\;2/3\right\}\right)$ algorithm versus the sampling ratio for the mSNR=40 dB 2D MRI image.

MRI is a typical medical inverse problem that can be solved well by CS [8]. In this experiment, we apply the proposed algorithm to real-world medical data. We investigate a simplified “dynamic MRI” problem [8] and use the high-quality MRI cardiac cine as the ground truth and select a spatio-temporal slice of 144×48 [28]. We construct the sparse matrix $\Psi \in R^{3N\times N}$ with a vertical concatenation of ‘*db1*’ and ‘*db2*’ orthogonal discrete wavelet bases with two levels of decomposition (‘*db1*’, ‘*db2*’, and N=2). Figure 12 presents the constructed MRI images using the SAITA-$L_{p}\left(p\in \left\{1/2,\;2/3\right\}\right)$ algorithm and $L_{p\;}\left(p\in \left\{1/2,\;2/3\right\}\right)$ algorithms. From the experiment results, we can see that the proposed SAITA algorithm can reconstruct the images perfectly, as shown in Figure 12b,d, while the conventional algorithms failed to restore the image in Figure 12a,c. The proposed multiple sub-dictionaries algorithm of p=2/3 obtained the best effect and the corresponding recovery SNR is 23.1872 dB. Next is the proposed algorithm of p=1/2 with recovery SNRs of 21.0189 dB. In Figure 13, we depict the RSNR of four algorithms versus different sampling ratios M/N. When p=1/2 and p=2/3, it can be observed that the proposed SAITA-$L_{p}$ algorithms can obtain a lager RSNR compared with the conventional single dictionary $L_{p}$ algorithms. The results also show that the algorithms of p=2/3 outperform the methods of p=1/2. That is to say, the $L_{2/3}$ regularization can exploit more prior knowledge than $L_{1/2}$ regularization in MRI.

## 6. Conclusions

In this paper, we propose a novel adaptive iteratively weighted thresholding algorithm (SAITA-$L_{p}$) based on the conventional $L_{1/2}$ and $L_{2/3}$ thresholding algorithm by incorporating the multiple sub-dictionary sparse representation strategy. We make the following conclusions from the above experiments:

(1) Using the proposed multiple sub-dictionary sparsifying transforms strategy, we construct a multiple sub-dictionary based $L_{p}$ regularization method to exploit more priori knowledge of images for the sparse image recovery problem. By multiplying by the proposed adaptive weighting parameter $\lambda_{d,p}^{t}=\frac{N_{d}}{{\left(\epsilon +{∥\Psi_{d}x∥}_{2}^{2}\right)}^{\frac{1-p}{2}}}$, we can gain more control of weighting the contribution of each sub-dictionary based regularizer. Experiments show that the proposed algorithms appear to perform better than the conventional single-dictionary algorithms, especially when the sampling rate is very low (e.g., 0.1~0.3);
(2) Compared with the $L_{1}$-norm regularization based work, the nonconvex $L_{p}(0<p<1)$-norm penalty can more closely approximate the $L_{0}$-norm minimization over the $L_{1}$-norm, which gives a sparser solution and needs fewer measurement data.
(3) In our experiments, we find that the recovery performances between the $L_{p}\;\left(p=1/2\right)$ and $L_{p}\left(p=2/3\right)$ are close, even when the corresponding p=2/3 algorithm can obtain a better performance over the p=1/2. Hence, a proper p need to be selected in practical applications.
(4) Moreover, the proposed SAITA-$L_{p}$ method also indicates that it is feasible to improve the recovery performance by exploiting the signal inner sparse structure and designing a proper sparse representation dictionary. Thus, it is beneficial to exploit the signal sparse structure with a dictionary learning method, which will be the subject of future work.
(5) The proposed SAITA-$L_{p}$ algorithm can be extended to other non-convex penalties include smoothly clipped absolute deviation (SCAD) and minimax concave penalty (MCP).
